# Gain-of-Signal Assays for Probing Inhibition of SARS-CoV-2 M^pro^/3CL^pro^ in Living Cells

**DOI:** 10.1128/mbio.00784-22

**Published:** 2022-04-26

**Authors:** Seyed Arad Moghadasi, Morgan A. Esler, Yuka Otsuka, Jordan T. Becker, Sofia N. Moraes, Constance B. Anderson, Srinivas Chamakuri, Christopher Belica, Chloe Wick, Daniel A. Harki, Damian W. Young, Louis Scampavia, Timothy P. Spicer, Ke Shi, Hideki Aihara, William L. Brown, Reuben S. Harris

**Affiliations:** a Department of Biochemistry, Molecular Biology and Biophysics, University of Minnesota, Minneapolis, Minnesota, USA; b Masonic Cancer Center, University of Minnesota, Minneapolis, Minnesota, USA; c Institute for Molecular Virology, University of Minnesota, Minneapolis, Minnesota, USA; d Department of Molecular Medicine, Scripps Research, The Scripps Research Molecular Screening Center, Jupiter, Florida, USA; e Department of Medicinal Chemistry, University of Minnesota, Minneapolis, Minnesota, USA; f Center for Drug Discovery, Department of Pathology & Immunology, Baylor College of Medicine, Houston, Texas, USA; g Department of Pharmacology and Chemical Biology, Baylor College of Medicine, Houston, Texas, USA; h Department of Biochemistry and Molecular Biology, Baylor College of Medicine, Houston, Texas, USA; i Howard Hughes Medical Institute, University of Minnesota, Minneapolis, Minnesota, USA; Ohio State University

**Keywords:** coronavirus, gain-of-signal cell-based systems, main protease (M^pro^/3CL^pro^), SARS-CoV-2 (SARS2), viral protease inhibitors

## Abstract

The main protease, M^pro^, of SARS-CoV-2 is required to cleave the viral polyprotein into precise functional units for virus replication and pathogenesis. Here, we report quantitative reporters for M^pro^ function in living cells in which protease inhibition by genetic or chemical methods results in robust signal readouts by fluorescence (enhanced green fluorescent protein [eGFP]) or bioluminescence (firefly luciferase). These gain-of-signal systems are scalable to high-throughput platforms for quantitative discrimination between M^pro^ mutants and/or inhibitor potencies as evidenced by validation of several reported inhibitors. Additional utility is shown by single M^pro^ amino acid variants and structural information combining to demonstrate that both inhibitor conformational dynamics and amino acid differences are able to influence inhibitor potency. We further show that a recent variant of concern (Omicron) has an unchanged response to a clinically approved drug, nirmatrelvir, whereas proteases from divergent coronavirus species show differential susceptibility. Together, we demonstrate that these gain-of-signal systems serve as robust, facile, and scalable assays for live cell quantification of M^pro^ inhibition, which will help expedite the development of next-generation antivirals and enable the rapid testing of emerging variants.

## INTRODUCTION

Viral proteases are proven targets for highly effective antiviral therapies, with leading examples being HIV-1 and HCV protease drugs (1–3). SARS-CoV-2 (SARS2) has two proteases, papain-like protease (PL^pro^, Nsp3) and main protease/3C-like protease (M^pro^, 3CL^pro^, Nsp5), which are responsible for 3 (Nsp1-4) and 11 (Nsp4-16) viral polyprotein cleavage events, respectively (4–7). These cleavage events are essential for the formation of the viral replicase complex and pathogenesis, and therefore, both of these SARS2 proteases are under intensive investigation for the development of drugs to combat the ongoing COVID-19 pandemic (8).

Since the first SARS pandemic in the early 2000s, several biochemical and structural biology studies have focused on M^pro^ and led to the identification of multiple small-molecule inhibitors (9–11). For most *in vitro* assays, fully mature M^pro^ is first purified from bacteria and subsequently treated with an orthologous protease to remove the purification tag and expose the native N and C termini, which are necessary for full M^pro^ catalytic activity (12, 13). Catalytic activity is measured by incubating M^pro^ with a peptide substrate containing a cleavage motif, typically corresponding to the N-terminal cognate cleavage site between Nsp4 and Nsp5. These peptides also have either an N and C terminal fluor-quench pair or a fluorescence resonance energy transfer (FRET) pair, and cleavage releases the covalent association and results in increased fluorescence or decreased FRET, respectively (13, 14). Inhibitor potency is measured by preincubating M^pro^ with compound concentrations and then peptide substrate is added to readout catalytic activity. These *in vitro* assays are critical for fundamental studies as well as for inhibitor identification and characterization. However, biochemical assays require M^pro^ to be fully mature and necessarily utilize *trans*-cleavage as a readout, and, therefore, such assays do not provide metrics for the earliest steps of infection, including M^pro^ folding, dimerization, and *cis*-cleavage from the larger viral polyprotein. Protease purifications are also challenging to scale-up for comparisons of dozens to hundreds of constructs (i.e., lab-designed mutants, naturally occurring variants, and/or enzymes from other coronavirus species).

Considerable effort has therefore also been invested in developing robust, safe, and scalable cellular systems for reporting M^pro^ activity. Cellular systems also add value by reflecting other key parameters of small-molecule development, including membrane permeability, metabolic liability, and off-target effects, including toxicity. A common live cell assay used currently is based on the FlipGFP concept (15–19). This assay relies on M^pro^-dependent cleavage of a linker domain separating β-strands 1 to 9 and β-strands 10 to 11 of GFP, which enables a conformational change (flip) and reconstitution of the full GFP β-barrel and fluorescence emission. However, this assay has yielded variable signal/noise ratios in multiple different labs (16–20). For instance, low sensitivity contributed to high-throughput sequencing (HTS) identification of compounds that reduce GFP signal independent of M^pro^ inhibition (17). An analogous cleavage-dependent assay has also been developed in which M^pro^ converts a circularized, inactive luciferase construct into a properly folded, active enzyme (19, 21). Although this assay has increased sensitivity compared to FlipGFP, it also has the potential to identify false-positive hits in screens due to a wide variety of other factors that can cause signal loss, including toxicity. Gain-of-signal assays have also been developed for M^pro^ inhibition (22, 23). However, these nanoluciferase-based assays also appear to lack sensitivity and seem to require high inhibitor concentrations for clear experimental readouts.

Here, we demonstrate highly sensitive and reproducible gain-of-signal assays for quantifying genetic or chemical inhibition of SARS2 M^pro^ activity in living cells, which utilize either enhanced green fluorescent protein (eGFP) fluorescence or luciferase luminescence as experimental readouts. Amino acid changes that disrupt M^pro^ function, including catalytic, allosteric, and dimerization mutants, elicit substantial increases in fluorescent or luminescent signal compared to the fully active wild-type enzyme. Similarly, treatment of cells expressing the wild-type M^pro^ reporter with known inhibitors of M^pro^ causes dose-responsive increases in signal, with the luciferase readout showing a 100-fold dynamic range, sensitivity into the low-nanomolar range, and adaptability to a 1,536-well plate format for HTS. This system was used to test the impact of amino acid changes surrounding the active site and identify a circulating variant with increased susceptibility to boceprevir but unchanged susceptibility to GC376, which could be explained by the flexibility of GC376 binding to M^pro^ in crystal structures. Testing of a broader panel of reported inhibitors underscores the high stringency of the assay for ruling out likely off-target molecules and the utility for rapid testing of variants of concern against clinical-grade antivirals. Together, these results illustrate the broad utility of our assay for aiding in the development of M^pro^ antiviral compounds and understanding the *cis*-acting determinants that impart M^pro^ activity.

## RESULTS

### M^pro^-inactivating mutants elicit increased reporter eGFP fluorescence.

These studies initiated with the goal of developing a cell line that would be able to report SARS2 infection analogous to established HIV-1 assays (24, 25). Reporter cell lines used for HIV-1 infectivity rely on the virally encoded *trans*-activator of transcription (Tat) to bind to an integrated HIV-1 long terminal repeat sequence to activate transcriptional elongation of a downstream reporter such as eGFP or firefly luciferase. As the transcriptional function of Tat relies on nuclear localization, we hypothesized that if Tat could be sequestered in the cytoplasm by an M^pro^-cleavable cytosolic membrane anchor such as the N-terminal myristoylation domain from the Src kinase, then M^pro^-catalyzed cleavage during infection would cause relocalization of Tat to the nucleus to activate expression of a reporter construct. However, before testing this concept with infectious virus, we sought to determine whether *cis-*cleavage of an in-frame M^pro^ would result in the anticipated relocalization phenotype.

**FIG 1:**
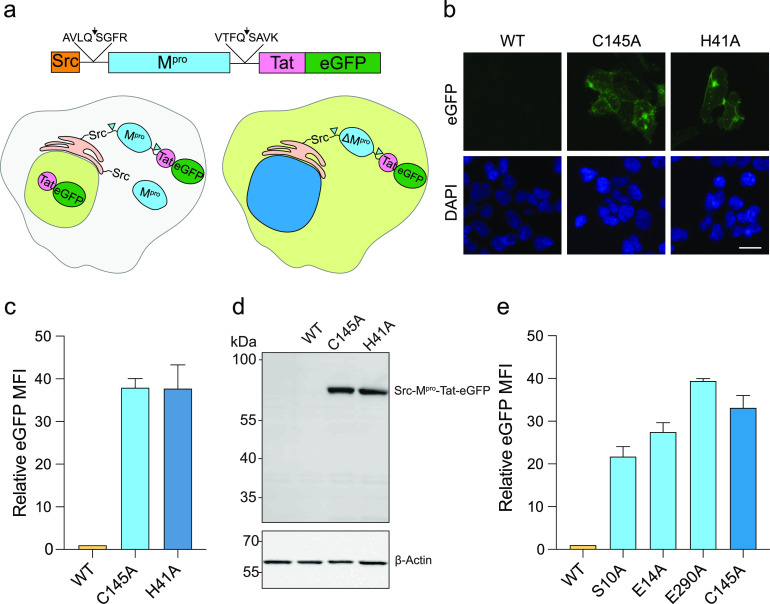
Gain-of-signal system for SARS-CoV-2 M^pro^ inhibition in living cells. (a) Schematic of the Src-M^pro^-Tat-eGFP wild-type (WT) reporter construct (see the text for details). M^pro^-catalyzed self-cleavage is predicted to result in Tat-eGFP nuclear localization, whereas cleavage inhibition causes cytoplasmic localization. (b) Representative fluorescence microscopy images of 293T cells expressing the indicated WT or catalytic mutant constructs (green). Hoechst staining (blue) shows the location of the nuclear compartment. Scale bar, 10 μm. (c) Bar graph of the eGFP mean fluorescence intensity (MFI) of the indicated catalytic mutant constructs in 293T cells 48 h post-transfection (mean ± SD of *n* = 3 biologically independent experiments). (d) Anti-eGFP immunoblot of the indicated M^pro^ constructs following 48 h expression in 293T cells. A parallel anti-β-actin blot was done as a loading control. (e) Bar graph of the eGFP MFI of the indicated dimer interface mutant constructs in 293T cells 48h post-transfection (mean ± SD of *n* = 3 biologically independent experiments).

Therefore, we constructed a chimeric protein consisting of an N-terminal myristoylation domain from Src kinase, the full M^pro^ amino acid sequence with cognate N- and C-terminal self-cleavage sites, HIV-1 Tat, and eGFP (to visualize localization) (Fig. 1a). Surprisingly, transfection of this wild-type (WT) construct into 293T cells failed to yield green fluorescence by microscopy or flow cytometry (Fig. 1b and c). However, an otherwise identical construct with a mutation of either residue comprising the catalytic dyad in M^pro^ (C145A or H41A) resulted in high levels of eGFP fluorescence, suggesting that proteolytic activity is responsible for the apparent lack of expression of the WT construct. In support of this interpretation, anti-GFP immunoblotting also indicated no reporter expression in cells expressing the WT construct; however, both catalytic mutants yielded a single band at the predicted molecular weight of the full-length Src-M^pro^-Tat-eGFP polyprotein (Fig. 1d).

Coronavirus M^pro^ is active as a homodimer, and mutations that disrupt dimerization also abrogate catalytic activity (26). Therefore, mutations that abolish dimerization should also result in increased fluorescence. Indeed, three single amino acid substitution mutations at the dimerization interface (S10A, E14A, and E290A) resulted in substantial increases in eGFP fluorescence to levels similar to those of the C145A catalytic mutant (Fig. 1e). These data confirm that M^pro^ disruption leads directly to higher eGFP signal and provide further support for the interpretation that protease activity and reporter activity are related inversely.

### Chemical inhibitors of M^pro^ trigger reporter activation and phenocopy genetic mutants.

**FIG 2:**
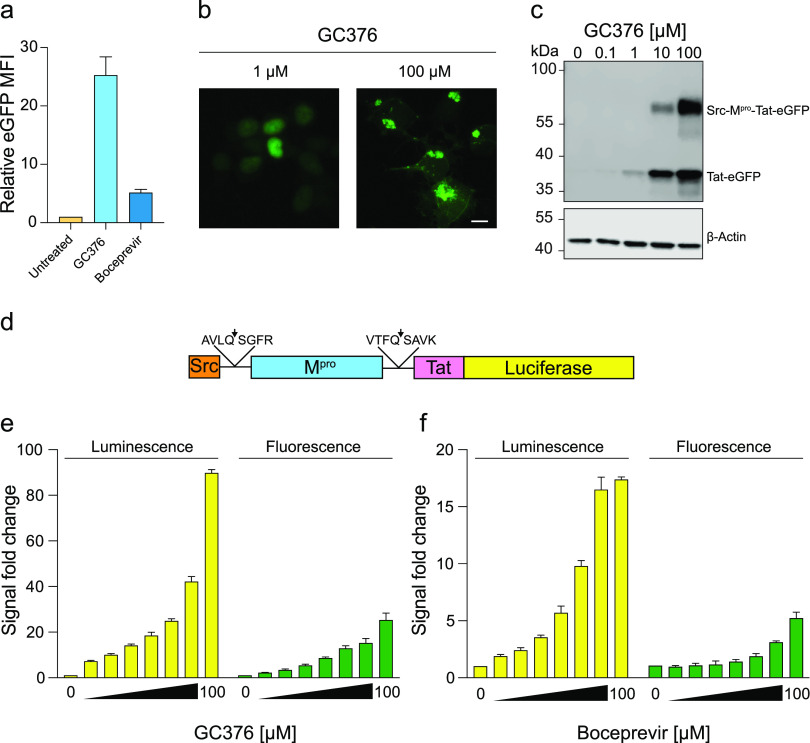
Small-molecule inhibitors of M^pro^ restore reporter signal. (a) Histogram of the eGFP MFI of 293T cells expressing the WT reporter and incubated with 100 μM GC376, 100 μM boceprevir, or DMSO (mean ± SD of *n* = 3 biologically independent experiments; data normalized to DMSO control). (b) Representative images of 293T cells expressing the WT reporter and treated with 1 or 100 μM GC376 (scale bar, 10 μm). (c) Anti-eGFP immunoblot showing differential accumulation of Tat-eGFP and Src-M^pro^-Tat-eGFP following incubation with the indicated amounts of GC376. A parallel anti-β-actin blot was done as a loading control. (d) Schematic of the Src-M^pro^-Tat-Luc wild-type (WT) reporter construct (see the text for details). (e and f) Histograms comparing the dose responsiveness of WT eGFP- and Luc-based reporter constructs to GC376 and boceprevir, respectively (2-fold dilution series beginning at 100 μM; mean ± SD of *n* = 3 biologically independent experiments with data normalized to DMSO control set to 1).

Multiple small-molecule inhibitors of M^pro^ have been described, including GC376 and boceprevir, with *in* vitro 50% inhibitory concentration (IC_50_) values of 0.03 to 0.19 μM and 1.6 to 8.0 μM, respectively (8, 27, 28). GC376 was developed against a panel of 3C and 3C-like cysteine proteases (29, 30), and boceprevir was developed as an inhibitor of the NS3 protease of hepatitis C virus (1, 31, 32). These small molecules have also been cocrystallized with SARS2 M^pro^, and their binding sites are well defined (33–35). We therefore next asked whether these compounds might mimic the genetic mutants described above and restore fluorescence activity of the WT construct. Treatment of cells with 100 μM GC376 caused a strong restoration of expression and eGFP fluorescence, whereas 100 μM boceprevir caused a comparatively modest increase in signal (Fig. 2a). Importantly, both compounds yielded striking signal increases above background levels in dimethyl sulfoxide (DMSO)-treated cells. The potency of GC376 was confirmed in dose-response experiments with both fluorescence microscopy and immunoblotting as experimental readouts (Fig. 2b and c).

At high concentrations of GC376 (100 μM), the subcellular localization of the WT construct phenocopies the C145A catalytic mutant with cytoplasmic membrane targeting due to the N-terminal myristoyl anchor (Fig. 2b). However, at lower concentrations (1 μM), the eGFP signal becomes predominantly nuclear, which is likely due to residual M^pro^ cleavage activity and import of the Tat-eGFP portion of the chimera into the nuclear compartment through the strong nuclear localization signal (NLS) of Tat (36) (Fig. 2b). Immunoblots showed corroborating results with the smaller Tat-eGFP fragment evident at low GC376 concentrations and shift toward the predominant band being the full-length Src-M^pro^-Tat-eGFP protein at high drug concentrations (Fig. 2c). To confirm that the lower band is the C-terminal cleavage product, mutation of the C-terminal cleavage site (Q306A) yields only the full-length uncleaved Src-M^pro^-Tat-eGFP band by immunoblotting extracts from cells treated with 10 μM GC376 (Fig. S1 in the supplemental material).

10.1128/mbio.00784-22.1 FIG S1 Accumulation of the shorter Tat-eGFP fragment requires a functional SARS2 M^pro^ C-terminal cleavage site. An anti-eGFP immunoblot of soluble extracts from cells expressing the WT Src-M^pro^-Tat-eGFP construct in comparison to a C-terminal cleavage site mutant (Q306A) following incubation with DMSO or 10 μM GC376. A parallel anti-β-actin blot was done as a loading control. Download FIG S1, PDF file, 0.4 MB. Copyright © 2022 Moghadasi et al. 2022 Moghadasi et al. https://creativecommons.org/licenses/by/4.0/ This content is distributed under the terms of the Creative Commons Attribution 4.0 International license.

### A luciferase-based reporter provides a more sensitive readout for M^pro^ inhibition.

To be able to detect lower-potency M^pro^ inhibitors, we next asked whether assay sensitivity might be increased by switching the readout from eGFP to firefly luciferase, which is capable of providing an enzyme-catalyzed signal amplification (Fig. 2d). As anticipated, a luciferase-expressing construct showed a substantial ~3-fold increase in signal across all tested concentrations for both GC376 and boceprevir (Fig. 2e and f). Moreover, application of a wide range of concentrations of GC376 to cells expressing the luciferase-based construct yielded an ~100-fold dynamic range in protease inhibition signal, with 200 nM compound triggering a 2-fold increase and 100 μM a 100-fold increase over background levels of luminescence normalized to 1 (Fig. S2a and b). These results demonstrate that the construct is compatible with a different readout and could likely be adapted for any reporter of choice, either fluorescent or enzymatic. This luciferase-based system can also be miniaturized to a 1,536-well plate format with highly reproducible signals due to M^pro^ inhibition (Z′ score of 0.70 using 10 μM GC376 as a positive control (Fig. S2c to e).

10.1128/mbio.00784-22.2 FIG S2 Sensitivity and miniaturization of Luc-based reporter system for M^pro^ inhibition. (a) GC376 dose responsiveness of the WT Luc-based reporter expressed in 293T cells. All values normalized to the luminescence of the DMSO-treated condition (mean ± SD shown for *n* = 3 parallel reactions for each condition). (b) Mean signal to background (S/B) ratios for the experiment shown in panel a showing a 100-fold dynamic range. (c) Schematic of the 1,536-well plate experimental setup in which Luc-based SARS2 M^pro^ reporter-expressing cells are subjected to dose-response testing with GC376 and boceprevir. A representative luminescent image is shown below captured using the ViewLux microplate imager. (d and e) Histograms comparing the 1,536-well format dose responsiveness of the Luc-based reporter to GC376 and boceprevir, respectively (mean ± SD of *n* = 4 biologically independent experiments). Download FIG S2, PDF file, 2.5 MB. Copyright © 2022 Moghadasi et al. 2022 Moghadasi et al. https://creativecommons.org/licenses/by/4.0/ This content is distributed under the terms of the Creative Commons Attribution 4.0 International license.

### Assay modularity allows testing of M^pro^ enzymes from different coronaviruses.

**FIG 3:**
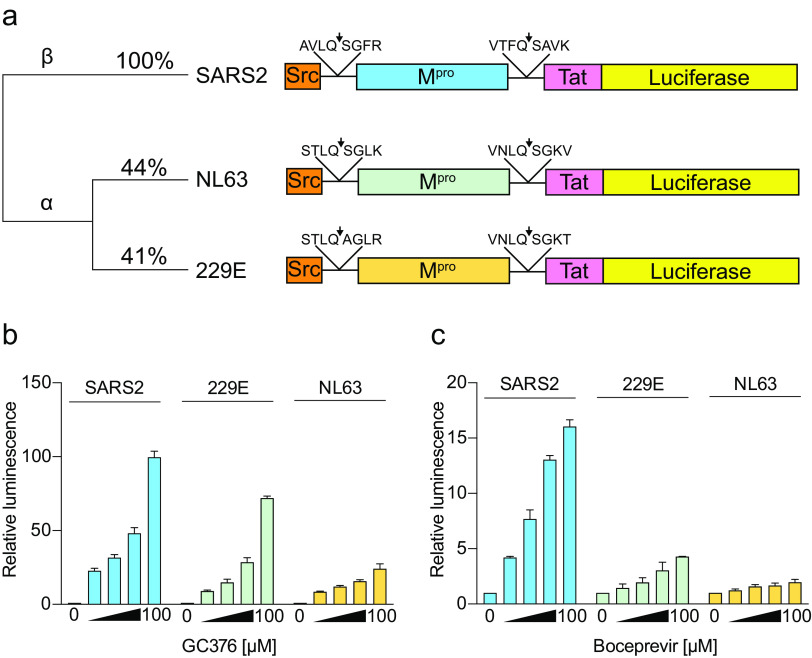
System modularity allows testing of M^pro^ enzymes from different coronaviruses. (a) Schematics of the indicated reporter constructs and phylogenetic relationships relative to SARS2 M^pro^. (b and c) Histograms comparing the dose responsiveness of the indicated Luc-based reporters to GC376 and boceprevir, respectively (2-fold dilution series beginning at 100 μM; mean ± SD of *n* = 3 biologically independent experiments).

As our chimeric construct reports inhibition of SARS2 M^pro^ catalytic activity, we next asked whether it might be compatible with proteases from distantly related coronaviruses. The M^pro^ enzymes from two human-infecting alpha-coronaviruses, HCoV-229E and HCoV-NL63, were therefore analyzed with flanking cognate cleavage sites in the context of the luciferase-based reporter (Fig. 3a). These two proteases have only 41 and 44% amino acid identity with SARS2 M^pro^ and, accordingly, are likely to have different susceptibilities to inhibitor treatments (schematics in Fig. 3a and alignment in Fig. S3). As above for the SARS2 M^pro^ reporter, low luminescent signals are observed following expression in human cells without inhibitor treatment, indicative of protease functionality (Fig. 3b, left). Moreover, despite extensive protease sequence divergence, GC376 is still able to cause a dose-responsive increase in luminescent signal over background for both 229E and NL63 M^pro^ (Fig. 3b, left). Interestingly, however, these two viral proteases are differentially susceptible to GC376, with maximal 72- and 24-fold signal increases, respectively, whereas SARS2 M^pro^ inhibition reaches a 100-fold signal increase. Lower reporter signals are observed following boceprevir treatment, with 229E showing a maximum increase of 4.3-fold and NL63 only 1.9-fold compared to 16-fold for SARS2 (Fig. 3b, right). This relatively high level of resistance to boceprevir is in line with *in vitro* studies reporting an IC_50_ of ~100 μM for boceprevir and NL63 M^pro^ (37). These results indicate that diverse coronavirus M^pro^ enzymes can function in the gain-of-signal system described here and, accordingly, can also be used for inhibitor testing. Thus, analogous adaptations to this system could be assets in future studies determining how narrow- or broad-acting a particular compound may be.

10.1128/mbio.00784-22.3 FIG S3 Crystal structures of GC376-bound SARS2 M^pro^ and rationally selected mutants. (a) Overview of the M^pro^ dimer, with GC376 bound in the active site shown in yellow sticks. (b) 2mFo-DFc electron density map contoured at 1.0 σ shown for the active site. (c) Superposition between our M^pro^-GC376 structure (yellow; PDB ID 7TGR) and complexes reported earlier. Structures and corresponding PDB ID numbers are color coded. (d) Multiple amino acid sequence alignment for the M^pro^ enzymes of the indicated coronaviruses (M^pro^ GenBank accession numbers, SARS2, QII57165.1; SARS1, QJE50587.1; HKU9, YP_001039970.1; murine hepatitis virus [MHV], NP_045299.2; Middle East respiratory syndrome-related coronavirus [MERS], QKF93417.1; 229E, AOG74782.1; NL63, AWK59972.1; IBV, UBM82395.1). SARS2 M^pro^ M49, P168, and Q189 are boxed, and identical residues are highlighted with blue. (e) Histogram showing background levels of luminescence for the indicated Luc-based SARS2 M^pro^ mutant constructs. Significantly increased luminescence indicates weakened protease activity (mean ± SD shown for *n* = 3 parallel experiments; *P* < 0.0001 for M49D versus WT and *P* < 0.01 for Q189N by unpaired *t* test). Download FIG S3, PDF file, 1.1 MB. Copyright © 2022 Moghadasi et al. 2022 Moghadasi et al. https://creativecommons.org/licenses/by/4.0/ This content is distributed under the terms of the Creative Commons Attribution 4.0 International license.

### Effects of M^pro^ single amino acid changes on inhibitor efficacy.

**FIG 4:**
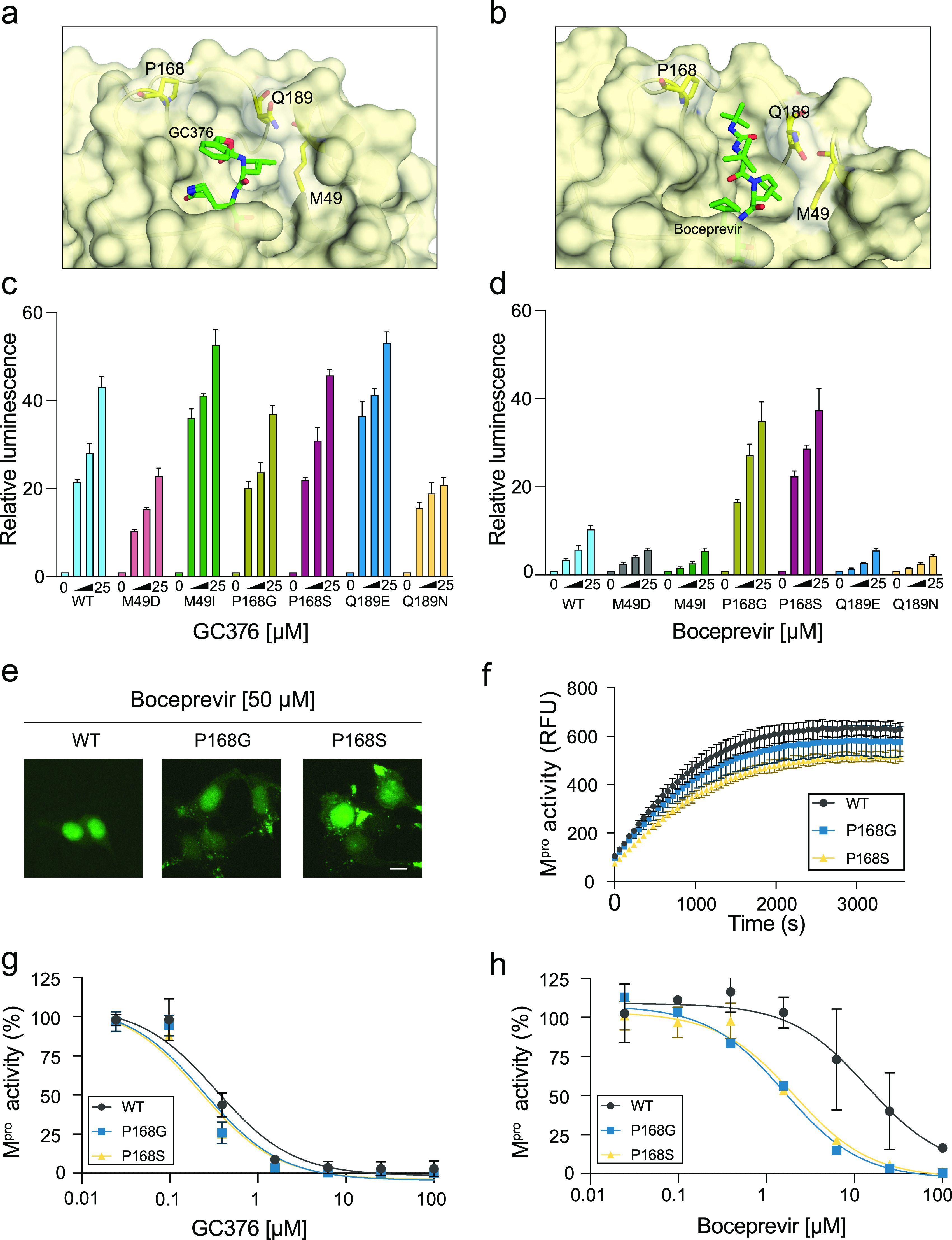
Inhibitor efficacies altered by single amino acid changes in SARS2 M^pro^. (a and b) Structures of SARS2 M^pro^ bound to GC376 and boceprevir, respectively, with mutable residues indicated (PDB ID 7TGR from this study and PDB ID 6WNP, respectively). (c and d) Histograms comparing the dose responsiveness of the indicated Luc-based reporters to GC376 and boceprevir, respectively (2-fold dilution series beginning at 25 μM; mean ± SD of *n* = 3 biologically independent experiments). (e) Representative images of 293T cells expressing the eGFP-based reporter with WT M^pro^, P168G, or P168S (50 μM boceprevir; scale bar, 10 μm). The mutant constructs elicit elevated fluorescence, including cytoplasmic accumulation, consistent with heightened drug sensitivity. (f) Time course of peptide cleavage by WT, P168G, and P168S SARS2 M^pro^ enzymes (mean ± SD shown for *n* = 2 independent runs). (g and h) Dose responsiveness of WT, P168G, and P168S SARS2 M^pro^ enzymes to GC376 and boceprevir, respectively (mean ± SD shown for *n* = 3 independent runs for each condition).

We next use the luciferase-based M^pro^ reporter system to ask how single amino acid changes might affect inhibitor efficacy. Guided by existing cocrystal structures of GC376 and boceprevir (13, 33–35), as well as a new GC376-M^pro^ X-ray structure from our collaborative studies (Fig. S3a to c), three residues, M49, P168, and Q189, were prioritized for dedicated experiments due to proximity to the active site and the fact that these positions vary between different coronaviruses (structural schematics in Fig. 4a and b; M^pro^ amino acid sequence alignments in Fig. S3d). M49D and Q189N had modest effects, decreasing the efficacy of GC376 by 2.1- and 1.8-fold, respectively (Fig. 4c). However, these two mutants also showed higher background signal than wild type (4-fold for M49D and 2-fold for Q189N), suggesting that the observed loss of compound efficacy may be due to weakened protease activity (Fig. S3e). Other amino acid changes at these positions, as well as substitutions at P168, had minimal effects on GC376 inhibition (Fig. 4c).

In stark contrast, M^pro^ P168G and P168S caused hypersensitivity to inhibition by boceprevir, yielding a 4-fold average increase in luciferase signal over wild type (Fig. 4d). A heightened susceptibility to boceprevir was also apparent using the eGFP-based assay, as both P168G and P168S show an increase in membrane-tethered cytosolic eGFP signal in comparison to the predominantly nuclear wild-type construct under the same conditions (Fig. 4e). The other amino acid substitutions, Q189N, Q189E, M49I, and M49D, each conferred a slight resistance to boceprevir in the luciferase-based system and no visible changes in the eGFP-based system (Fig. 4d; data not shown).

To further investigate the apparent heightened M^pro^ susceptibility to boceprevir, WT, P168G, and P168S proteins were expressed in Escherichia coli, purified to yield fully mature recombinant enzymes, and compared biochemically using a quenched fluorescent peptide substrate with a consensus cleavage site. Each mutant showed WT-like substrate cleavage kinetics and also a WT-like response to inhibition by GC376 (Fig. 4f and g) (IC_50_ values, WT, 0.34 μM; P168G, 0.25 μM; P168S, 0.23 μM). In contrast, boceprevir inhibited wild-type M^pro^ with an IC_50_ of 14 μM, and P168G and P168S showed approximately 10-fold lower IC_50_ values of 1.6 μM and 2.1 μM, respectively, consistent with the cell-based results above (Fig. 4h). The selective change in susceptibility to boceprevir and not GC376 can be explained by the binding pose of GC376 in our X-ray crystal structure in which the phenyl ring is able to point out into solvent and avoid being influenced by side chain changes at residues that contribute to the S4 subsite, including P-, G-, and S168 (Fig. 4a and Fig. S5). It is therefore notable that P168S is the most common amino acid variant at M^pro^ position 168 in circulating SARS2 isolates (*n* = 400 on 26 February 2022; GISAID database https://www.gisaid.org/). P168G has yet to be observed in circulating isolates, likely because this substitution requires the simultaneous occurrence of two different nucleotide changes. Taken together, we propose that P168 single amino acid substitutions increase the flexibility of the M^pro^ active site and improve binding to boceprevir but not to GC376. Most importantly, these results highlight the usefulness of the luciferase-based system described here for rapidly and quantitatively comparing the drug susceptibilities of M^pro^ amino acid variants (natural or structure guided), and they also underscore the need to carefully compare results from assays in living cells and biochemical assays *in vitro* with purified enzymes.

10.1128/mbio.00784-22.5 FIG S5 Evaluating a general mechanism of expression inhibition caused by M^pro^. (a) Cell viability in response to GC376 treatment in cells transfected with reporter relative to a DMSO-treated control (mean ± SD shown for *n* = 3 parallel reactions). (b) Schematic of a two-color reporter construct encoding Src-M^pro^-Tat-eGFP driven from a CMV promoter and mCherry driven from an SV40 promoter (pA, polyadenylation signal) and signal fold change of eGFP and mCherry in response to GC376 as measured by flow cytometry with background signal normalized to 1 (mean ± SD shown for *n* = 3 parallel reactions). (c) Schematic of the construct with an antisense U6 promoter (RNA PolIII) driving expression of a firefly luciferase fragment in addition to the sense-oriented CMV-driven Src-Mpro-Tat-eGFP (pT = RNA PolIII transcription termination sequence) with quantification of Src-Mpro-Tat-eGFP mRNA relative to the U6-driven transcript in the cytosolic fraction of 293T cells transfected with either WT plasmid (±50 μM GC376) or the C145A mutant plasmid. The RNA ratio under WT conditions is normalized to 1 for comparison (mean ± SD shown for *n* = 3 parallel reactions). Download FIG S5, PDF file, 0.3 MB. Copyright © 2022 Moghadasi et al. 2022 Moghadasi et al. https://creativecommons.org/licenses/by/4.0/ This content is distributed under the terms of the Creative Commons Attribution 4.0 International license.

### Comparative analyses of reported M^pro^ inhibitors.

While some reported SARS2 M^pro^ inhibitors have gone through rigorous characterization and structure-activity relationships have been established, other candidates have been identified through high-throughput screens in drug repurposing efforts, and on-target efficacies remain controversial (9, 38–46). We therefore used our luciferase-based gain-of-signal assay to directly compare candidate inhibitors and help shed light on this important and rapidly growing area.

**FIG 5:**
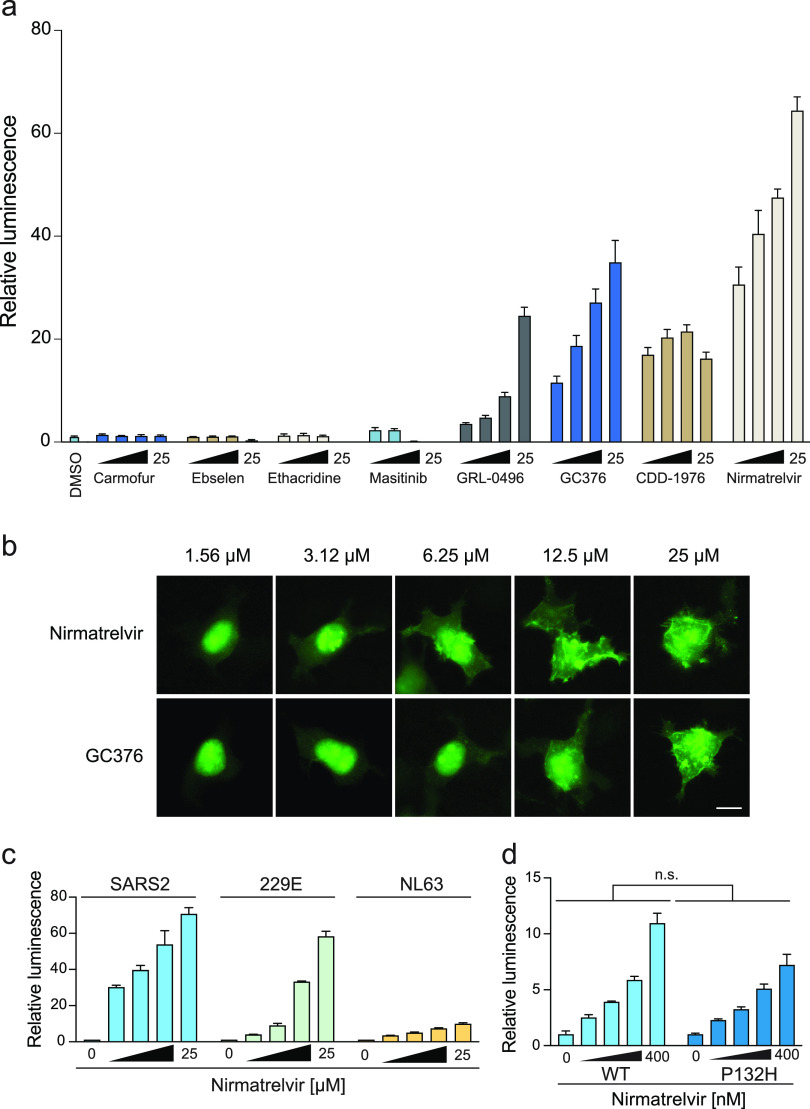
Comparative analyses of reported SARS2 M^pro^ inhibitors. (a) Dose responsiveness of the WT SARS2 M^pro^ construct expressed in 293T cells to the indicated compounds (2-fold dilution series beginning at 25 μM). All values normalized to the luminescence of the DMSO-treated condition (mean ± SD shown for *n* = 3 parallel reactions for each condition). Higher concentrations of CD1976 limit signal accumulation due to toxicity. (b) Representative images of 293T cells expressing the eGFP-based reporter treated with nirmatrelvir and GC376. Scale bar, 10 μm. (c) Dose responsiveness of the indicated M^pro^ enzymes to nirmatrelvir (2-fold dilution series beginning at 25 μM; mean ± SD of *n* = 3 biologically independent experiments). (d) Dose responsiveness of SARS2 P132 (WT) and H132 (Omicron) M^pro^ enzymes to nirmatrelvir (2-fold dilution series beginning at 400 nM; mean ± SD of *n* = 3 biologically independent experiments; *P* = 0.61 by unpaired Student's *t* test).

As above, GC376 inhibits SARS2 M^pro^, causes increased reporter expression, and provides a clear metric for inhibitor cross-comparisons (Fig. 5a). The recently reported compound, PF-07321332 (nirmatrelvir) (40), performs the best in our assay by producing a clear dose response and the highest reporter signals at all tested concentrations (Fig. 5a). GC376, nirmatrelvir, and boceprevir (above) are all peptide-based inhibitors. It is therefore notable that two nonpeptide chemotypes also tested positive. CDD-1976, identified recently in a DNA-encoded library screen (39), shows strong inhibitory activity at lower concentrations and blunted activity at higher concentrations due to cytotoxicity (Fig. 5a). GRL-0496, identified originally as a SARS1 M^pro^ inhibitor (9), also yields a clear positive dose response and no cytotoxicity (Fig. 5a). The greater potency of nirmatrelvir in comparison to GC376 was further validated by imaging with the eGFP-based assay, which shows that nirmatrelvir inhibits *cis-*cleavage at lower concentrations than GC376, as inferred by cytoplasmic fluorescence (Fig. 5b).

In contrast to the 5 different compounds that inhibited SARS2 M^pro^ and triggered reporter expression to various degrees, 4 other reported compounds tested negative at all concentrations (Fig. 5a). Carmofur and ebselen were identified in high-throughput screens *in vitro*, shown to elicit antiviral activity in cell culture models, and have been cocrystallized with pure enzyme (42, 43, 47, 48). Ethacridine was identified by high-throughput screening using a M^pro^ FlipGFP live cell assay; however, its mechanism of antiviral action is likely through viral particle inactivation (41). Last, masitinib was identified recently as a pan-coronavirus inhibitor in a drug-repurposing screen, assessed using the aforementioned FlipGFP system and a related luciferase-based biosensor, and shown to elicit strong antiviral activity (45). To ensure these results are not due to compound issues, the purity and molecular weight of all negative-testing compounds were confirmed by high-pressure liquid chromatography (HPLC) (Table S1). As our assay assesses on-target activity in living cells, which could be influenced by many different mechanisms, we are not challenging the likelihood that these inhibitors inhibit M^pro^ catalysis *in vitro*. Nevertheless, despite these initial reports, the clear negative results using our gain-of-signal system call into question the mechanism(s) of action of these putative M^pro^ inhibitors and suggest that the reported antiviral activities of these compounds are likely to be nonspecific.

10.1128/mbio.00784-22.8 TABLE S1 Inhibitor sources. Download Table S1, PDF file, 0.08 MB. Copyright © 2022 Moghadasi et al. 2022 Moghadasi et al. https://creativecommons.org/licenses/by/4.0/ This content is distributed under the terms of the Creative Commons Attribution 4.0 International license.

Last, because nirmatrelvir was approved recently as a first-in-class SARS2 M^pro^ inhibitor (https://www.fda.gov/media/155049/download), we assessed the broader-spectrum potential of this drug using distantly and closely related coronavirus variants. Interestingly, nirmatrelvir showed much stronger activity against the M^pro^ enzyme of 229E in comparison to that of NL63, despite the fact that these two alpha-coronavirus enzymes are closely related (71% identity) (Fig. 5c). These results suggest that this compound may have the potential for broader-spectrum activity, but sequence differences could exist and provide pathways to resistance. Finally, the emerging variant of concern, Omicron, which has a single amino acid change in M^pro^ (P132H), retains sensitivity to nirmatrelvir even at low-nanomolar concentrations, which bodes well for clinical application (Fig. 5d).

## DISCUSSION

Here, we describe two gain-of-signal assays for measuring inhibition of coronavirus M^pro^ by chemical inhibition or mutational inactivation. The original reporter, Src-M^pro^-Tat-eGFP, shows very low protein expression, which can be rescued by mutating residues required for protease function (i.e., catalytic dyad or dimer interface residues). These genetic results suggested that reporter signal is inversely related to M^pro^ function and that chemical inhibitors may be similarly effective at recovering signal. Indeed, SARS2 M^pro^ inhibitors, GC376 and boceprevir, both cause a dose-dependent restoration of reporter signal, with the former compound being more effective, consistent with prior work (33). These inhibitors were further used to demonstrate assay robustness and modularity, with firefly luciferase providing a higher signal than eGFP and other coronaviral main proteases able to function in place of the SARS2 enzyme. The utility of the more sensitive luciferase-based reporter is further demonstrated by performing a comparative analysis of a panel of reported SARS2 M^pro^ inhibitors. Compounds that test negative in this gain-of-signal system, including masitinib (45), are likely to be nonspecific. The most effective M^pro^ inhibitor reported to date, nirmatrelvir (40), elicits the highest signals in our systems and provides a clear benchmark for additional drug development efforts.

As for therapies targeting HIV-1 and HCV proteases (49, 50), drug resistance mutations are also likely to become a concern in treating SARS2 infections. The luciferase-based gain-of-signal assay provides a facile system for the systematic comparison of amino acid variants. This application is demonstrated here in multiple ways. First, the M^pro^ enzymes of distantly related coronaviruses also function in this system (Fig. 3), and future studies may be used to map amino acids responsible for differential drug responses. Second, differential susceptibility to GC376 and boceprevir is shown for structure-informed single amino acid substitution mutants (Fig. 4). This is best evidenced by two mutants, P168G and P168S, that confer a 4-fold increased susceptibility to boceprevir in the gain-of-signal assay but no change in GC376 susceptibility. These results are likely to be intrinsic to the enzyme because recombinant versions also show similarly heightened susceptibilities *in vitro*. P168S is also notable as a naturally occurring variant of SARS2 M^pro^. Third, a current SARS2 variant of concern, Omicron, has a single amino acid change in M^pro^ (P132H) that does not significantly change the potency of nirmatrelvir (Fig. 5). This result is encouraging for clinical applications and, together with the aforementioned results, further underscores the utility of the assay described here for rapidly testing single amino acid variants as they continue to emerge.

The finding that amino acid changes at P168 alter susceptibility to boceprevir but not GC376 has implications for designing next-generation M^pro^ inhibitors. The majority of crystal structures, including ours, show the phenyl ring of GC376 projecting outward into solvent, in contrast to a minority with a flatter binding pose and the ring occupying the S4 subsite (Fig. S3c in the supplemental material). These structural results, coupled with our inhibition data using P168 variants, indicate that conformationally flexible inhibitors such as GC376 are less likely to be affected by point mutations in and around the ligand binding site. As many resistance mutations can be explained by side chain clashes with inhibitors, compounds such as GC376 that can adopt multiple conformations may elicit higher barriers to resistance. A precedent for this design principle is the development of “fleximer” nucleoside analogues in which the bicyclic purine ring is split by a rotatable bond into two halves, which provide conformational freedom (51). Another example is the HIV-1 drug tenofovir, which has an intrinsic flexibility that allows it to resist some single amino acid changes (51). Taken together, increasing the flexibility of next-generation M^pro^ inhibitors may help increase the barrier to developing drug resistance.

A wide variety of assays will be needed to continue to advance our fundamental understanding of SARS2 nonstructural proteins, including M^pro^, and develop additional antiviral drugs. The cell-based gain-of-signal assay described here may be useful for high-throughput screening and, more importantly, also as a bridge between robust biochemical assays *in vitro* and more complex biosafety level 3 (BSL3) experiments requiring pathogenic viral isolates. Gain-of-signal assays have advantages over loss-of-signal assays because toxic and/or off-target compounds are less likely to score positive. For instance, compounds such as carmofur, ebselen, ethacridine, and masitinib, which were identified in loss-of-signal assays (41, 42, 45), would not be designated M^pro^ inhibitors using the system described here. The system described here may also be useful for identifying and studying M^pro^ dimerization inhibitors because it provides a model for coronavirus polyprotein maturation in which the viral protease must likely first dimerize before it can catalyze its own excision and undertake *trans*-cleavage events necessary for generating other nonstructural proteins. In comparison, biochemical assays require a preformed active protease dimer and are less likely to identify dimerization inhibitors in screens.

Finally, although we have systematically shown that both genetic and chemical inhibition of M^pro^ catalytic activity causes increased signal, the exact mechanism behind our reporter system is not known. However, several hints have emerged as to what may underlie the observed phenotypes. First, the C-terminal cleavage product that contains the eGFP or luciferase reporter protein is stable, making it unlikely that proteolytic cleavage produces an otherwise cryptic degron that leads to loss of signal. Second, a clear decrease in expression of the entire reporter construct polyprotein occurs following reporter transfection, suggesting a more general mechanism. This possibility is supported by time course experiments showing similar luciferase signal accumulation for inhibitor-treated and untreated cells until 24 h post-transfection when signals bifurcate with GC376-treated cells continuing to yield higher signal and untreated cells showing a plateau (Fig. S4a). Although it may be simple to assume that general cytotoxicity caused by M^pro^ could explain this phenotype, cell viabilities remain consistent throughout 48 h of incubation with a wide range of GC376 concentrations (Fig. S5a). A more global mechanism is also supported by increased signal following GC376 treatment of a secondary mCherry reporter driven by another promoter on the same plasmid as Src-M^pro^-Tat-eGFP (Fig. S5b). Finally, we tested the possibility that our phenotype may be occurring at the mRNA level by placing a U6 promoter (RNA Pol III)-driven noncoding fragment into the antisense direction of the same vector as an internal control (Fig. S5c). Reverse transcriptase quantitative PCR (RT-qPCR) quantification of the cytoplasmic pool of RNAs in this experiment shows that, relative to the wild-type M^pro^-expressing conditions, both catalytically inactive M^pro^ and inhibition by GC376 cause a >10-fold increase in the ratio of Src-M^pro^-Tat-eGFP mRNA relative to the U6 control RNA (Fig. S5c). Together, these results suggest a mechanism in which M^pro^ activity somehow suppresses the accumulation of reporter mRNA in transfected cells. However, such a mechanism may be multifactorial because overexpressed M^pro^ is known to cleave many different cellular proteins (52–55) in addition to authentic *cis*- and *trans*-viral polyprotein substrates. Regardless of the precise mechanism, the eGFP- and luciferase-based gain-of-signal reporter systems described here constitute robust assays for additional studies on coronavirus M^pro^ functionality and chemical inhibition.

10.1128/mbio.00784-22.4 FIG S4 Kinetics of reporter expression levels. (a) Representative time course experiment showing luciferase signal accumulation of 293T cells transfected with the WT Luc-based SARS2 M^pro^ reporter construct and treated with GC376 or DMSO for the indicated duration (mean ± SD shown for *n* = 3 parallel reactions per condition; **, *P* < 0.005; ***, *P* < 0.0005 by unpaired student’s *t* test). (b) Immunoblot time course of 293T cells transfected with eGFP-based reporter using a monoclonal anti-M^pro^ antibody and an anti-α-tubulin antibody as a loading control. Download FIG S4, PDF file, 0.6 MB. Copyright © 2022 Moghadasi et al. 2022 Moghadasi et al. https://creativecommons.org/licenses/by/4.0/ This content is distributed under the terms of the Creative Commons Attribution 4.0 International license.

## MATERIALS AND METHODS

### Plasmid constructs.

Nsp5, Tat, and eGFP coding sequences were amplified from existing vectors and fused using overlap extension PCR (Fig. S6 in the supplemental material). The final reaction added the 5′-myristolation sequence from Src and HindIII and NotI sites for restriction and ligation into similarly cut pcDNA5/TO (Thermo Fisher Scientific; catalog no. V103320). Wild-type and C145A catalytic mutant Nsp5 were amplified from pLVX-EF1alpha-nCoV2019-nsp5-2xStrep-IRES-Puro (55) using 5′-GTG-GGT-CAT-CTA-TCA-CCT-CAG-CTG-TTT-TGC-AGT-CTG-GTT-TTA-GGA-AAA-TGG-CGT-TCC-3′ and 5′-CCC-CCT-GAC-CCG-GTA-CCC-TTG-ATT-GTT-CTT-TTC-ACT-GCA-CTC-TGG-AAA-GTG-ACC-CCA-CTG-3′. The sequence encoding HIV-1 Tat residues 1 to 72 was amplified from an HIV-1 BH10 full molecular clone (56) using 5′-AGA-ACA-ATC-AAG-GGT-ACC-GGG-TCA-GGG-GGC-AGC-GGA-GGG-ATG-GAG-CCA-GTA-GAT-CCT-AGA-3′ and 5′-GGT-GGC-GAT-GGA-TCC-CGG-CTG-CTT-TGA-TAG-AGA-AAC-TTG-ATG-AGT-CT-3′. The eGFP coding sequence was amplified from pcDNA5/TO-A3B-eGFP (57) using 5′-AGA-CTC-ATC-AAG-TTT-CTC-TAT-CAA-AGC-AGC-CGG-GAT-CCA-TCG-CCA-CC-3′ and 5′-GAC-TCG-AGC-GGC-CGC-TTT-ACT-TGT-ACA-GCT-CGT-CCA-T-3′. The Src myristoylation sequence (58) was added using 5′-AAG-CTT-GCC-ACC-ATG-GGC-AGC-AGT-AAG-AGT-AAA-CCG-AAA-GAT-GGA-GGC-GGT-GGG-TCA-TCT-ATC-ACC-TCA-GCT-3′ and the eGFP reverse primer. Sanger sequencing confirmed the integrity of all constructs. The analogous firefly luciferase construct was created by amplifying the firefly luciferase open-reading from the pGL3-Basic firefly luciferase vector (catalog no. E1751; Promega) using 5′-GCA-GCC-GGG-ATC-CAT-CGC-CAC-CGA-AGA-CGC-CAA-AAA-CAT-AAA-GAA-AGG-CC-3′ and 5′-TCG-AGC-GGC-CGC-TTT-ACA-ATT-TGG-ACT-TTC-CGC-CCT-TCT TG-3′ and subcloned into the Src-M^pro^-Tat-eGFP construct by restriction ligation using the BamHI and NotI cut sites flanking eGFP (Fig. S7). All mutants were generated by site-directed mutagenesis using the primers listed in Table S2 and confirmed by Sanger sequencing.

10.1128/mbio.00784-22.6 FIG S6 Amino acid and nucleotide sequences of the Src-M^pro^-Tat-eGFP construct. (a) Domain descriptions and corresponding amino acid sequences with every other domain shaded gray to facilitate comparison. (b) Complete nucleotide sequence of the Src-M^pro^-Tat-eGFP construct from the HindIII 5′ restriction site to the NotI 3′ restriction site. The sequence is gray shaded to match the protein coding domains detailed above in panel a. The DNA sequences for Src and M^pro^ are codon optimized for expression in human cells. Download FIG S6, PDF file, 0.2 MB. Copyright © 2022 Moghadasi et al. 2022 Moghadasi et al. https://creativecommons.org/licenses/by/4.0/ This content is distributed under the terms of the Creative Commons Attribution 4.0 International license.

10.1128/mbio.00784-22.7 FIG S7 Amino acid and nucleotide sequences of the Src-M^pro^-Tat-Luc construct. (a) Domain descriptions and corresponding amino acid sequences with every other domain shaded gray to facilitate comparison. (b) Complete nucleotide sequence of the Src-M^pro^-Tat-Luc construct from the HindIII 5′ restriction site to the NotI 3′ restriction site. The sequence is gray shaded to match the protein coding domains detailed above in panel a. The DNA sequences for Src and M^pro^ are codon optimized for expression in human cells. Download FIG S7, PDF file, 0.2 MB. Copyright © 2022 Moghadasi et al. 2022 Moghadasi et al. https://creativecommons.org/licenses/by/4.0/ This content is distributed under the terms of the Creative Commons Attribution 4.0 International license.

10.1128/mbio.00784-22.9 TABLE S2 Site-directed mutagenesis and qPCR primers. Download Table S2, PDF file, 0.01 MB. Copyright © 2022 Moghadasi et al. 2022 Moghadasi et al. https://creativecommons.org/licenses/by/4.0/ This content is distributed under the terms of the Creative Commons Attribution 4.0 International license.

The two-color Src-M^pro^-Tat-eGFP construct was generated by amplifying the mCherry coding sequence using 5′-TTT-TTT-GGA-GGC-CTA-GGC-TTT-TGC-AAA-AAG-GCC-ACC-ATG-GTG-AGC-AAG-GGC-GAG-3′ and 5′-CAA-GCT-CCC-GGG-AGT-TAC-TTG-TAC-AGC-TCG-TCC-ATG-CC-3′ and subcloning this fragment into the AvrII and SmaI sites downstream of the simian virus 40 (SV40) promoter in the parental Src-M^pro^-Tat-eGFP construct. The U6-driven reporter RNA construct was generated by overlap extension PCR of DNA fragments, which included the U6 promoter amplified using 5′-CAG-ATA-TAC-GCG-TCC-CCA-GTG-GAA-AGA-CGC-G-3′ and 5′-CCT-TTC-TTT-ATG-TTT-TTG-GCG-TCT-TCC-GGT-GTT-TCG-TCC-TTT-CCA-CAA-GAT-ATA-TAA-AGC-3′ and a small fragment of luciferase amplified using 5′- GAA-GAC-GCC-AAA-AAC-ATA-AAG-AAA-GGC-C-3′ and 5′-ACT-ATT-AAT-AAC-TAG-TCA-ATA-ATC-AAT-GTC-ATT-CAT-AGC-TTC-TGC-CAA-CCG-AAC-3′. The combined fragment was subcloned into the Src-M^pro^-Tat-eGFP construct by restriction and ligation into the MluI site upstream of the cytomegalovirus (CMV) promoter.

### Cell culture and reporter assays.

293T cells were maintained at 37°C and 5%CO_2_ in RPMI 1640 (Gibco; catalog no. 11875093) supplemented with 10% fetal bovine serum (Gibco; catalog no. 10091148) and penicillin-streptomycin (Gibco; catalog no. 15140122). 293T cells were seeded in a 24-well plate at 1.5 × 10^5^ cells/well and transfected 24 h later with 200 ng of the wild-type or mutant chimeric reporter construct (TransIT-LT1; Mirus; catalog no. MIR2304). Forty-eight hours post-transfection, cells were washed twice with phosphate-buffered saline (PBS) and resuspended in 500 μL PBS. One-fifth of the cell suspension was transferred to a 96-well plate, mixed with TO-PRO3 ReadyFlow reagent for live/dead staining per manufacturer’s protocol (Thermo Fisher Scientific; catalog no. R37170), incubated at 37°C for 20 min, and analyzed by flow cytometry (BD LSRFortessa). The remaining four-fifths of the cell suspension was pelleted, resuspended in 50 μL PBS, mixed with 2× reducing sample buffer, and analyzed by immunoblotting (below).

For luciferase-based assays, 3 × 10^6^ cells were seeded in a 10-cm dish and transfected 24 h later with 2 μg of the Src-M^pro^-Tat-fLuc construct. Four hours post-transfection, cells were washed once with PBS-EDTA, trypsinized, resuspended, and counted. Cells were diluted to yield a suspension containing 4 × 10^5^ cells/mL, and 50 μL of the suspension was plated into a 96-well plate with 50 μL of media containing 2× the desired drug concentration yielding a final 1× drug concentration and 2 × 10^4^ cells/well. Forty-four hours after plating into 96-well plates, medium was removed, and 50 μL of Bright-Glo reagent (catalog no. E2610; Promega) was added, followed by a 5-min incubation before transferring the Bright-Glo and cell lysate into a white flat 96-well plate for measuring luminescence on a Tecan Spark plate reader. Cells were prepared in an identical manner as the luciferase assay for 3-(4,5-dimethylthiazol-2-yl)-5-(3-carboxymethoxyphenyl)-2-(4-sulfophenyl)-2H-tetrazolium (MTS) assays, and cell viability was assessed using the manufacturer’s protocol (Promega; catalog no. G1111).

### Protease inhibitors.

Compounds were purchased as powders from the commercial vendors listed in Table S1 and resuspended in DMSO to stock concentrations of 10 mM. CDD-1976 was provided by the Young lab (39). The integrity (purity and molecular weight) of all compounds that tested negative in the system described here was confirmed by HPLC (Table S1).

### Fluorescent microscopy.

Fifty thousand 293T cells were plated in a 24-well plate and allowed to adhere overnight. The next day, cells were transfected with 150 ng of each plasmid. Images were collected 48 h post-transfection at ×10 magnification using a the Cytation 5 multimode imager (Biotek).

### Immunoblots.

Whole-cell lysates in 2× reducing sample buffer (125 mM Tris-HCl, pH 6.8, 20% glycerol, 7.5% SDS, 5% 2-mercaptoethanol, 250 mM dithiothreitol [DTT], and 0.05% bromophenol blue) were denatured at 98°C for 15 min, fractionated using SDS-PAGE (4 to 20% Mini-Protean gel; Bio-Rad; catalog no. 4568093), and transferred to a polyvinylidene difluoride (PVDF) membrane (Millipore; catalog no. IPVH00010). Immunoblots were probed with mouse anti-GFP (1:10,000; JL-8, Clontech; catalog no. 632380) or rabbit anti-SARS-CoV-2 3C-like protease (1:1,000; Cell Signaling; catalog no. 51661) and rabbit anti-β-actin (1:10,000; Cell Signaling; catalog no. 4967) or mouse anti-α-tubulin (1:10,000; Sigma; catalog no. T5168) followed by goat/sheep anti-mouse IgG IRDye 680 (1:10,000; LI-COR; catalog no. 926-68070) or goat anti-rabbit IgG-horseradish peroxidase (HRP; 1:10,000; Jackson Laboratory; catalog no. 111-035-144). HRP secondary antibody was visualized using the SuperSignal West Femto maximum sensitivity substrate (Thermo Fisher; catalog no. PI34095). Images were acquired using the LI-COR Odyssey Fc imaging system.

### Protein preparation.

An E. coli codon-optimized synthetic gene for SARS2 M^pro^ (Twist Bioscience) was cloned into the BsaI restriction site of pE-SUMO vector (LifeSensors) via golden gate assembly. Expression plasmids for M^pro^ P168G and P168S were generated by site-directed mutagenesis. All plasmids were verified by Sanger DNA sequencing. A single colony of E. coli strain BL21(DE3) transformed with each expression plasmid was grown overnight to saturation in 25 mL LB medium supplemented with 100 μg mL^−1^ carbenicillin. The starter culture was then used to inoculate 3 L of ZYP-5052 auto-induction medium (59) supplemented with 100 μg mL^−1^ ampicillin divided across 9 baffled 2-L shake flasks. The bacterial cells were grown at 37°C for 4 h prior to lowering the temperature to 18°C and incubating for an additional 20 h. The cells were pelleted, resuspended in 20 mM Tris, pH 8.0, 500 mM NaCl, 5 mM β-mercaptoethanol, and 5 mM imidazole and lysed by sonication. M^pro^ protein was captured from cleared lysate using a nickel-nitrilotriacetic acid affinity column and eluted by a linear concentration gradient of imidazole. The eluted protein was treated overnight with the SUMO protease Ulp1 to remove the N-terminal His_6_-SUMO tag, which reveals the native N terminus of M^pro^. The cleaved protein was concentrated by ultrafiltration and further purified by size exclusion chromatography (SEC) on a Superdex 75 pg column operating with 20 mM Tris-HCl, pH 8.0, 150 mM NaCl, and 1 mM dithiothreitol (DTT). The peak fractions of SEC showing single band for M^pro^ in SDS-PAGE were pooled and concentrated to 12 mg mL^−1^ as determined by UV absorbance measured on a NanoDrop 8000 spectrophotometer and flash frozen in liquid nitrogen for storage at −80°C.

### Biochemical M^pro^ activity assays.

Biochemical activity of M^pro^ was analyzed using a quenched fluorescent peptide substrate DABCYL-KTSAVLQ↓SGFRKM-EDANS (Bachem; catalog no. 4045664), which has been used in multiple recent studies (33, 34, 60). M^pro^ cleavage between Gln and Ser liberates fluorescence, which was quantified by excitation and emission at 350 and 490 nm, respectively. The reactions were carried out in Greiner Bio-One 96-well chimney plates with 10 μM substrate, 200 nM M^pro^, 20 mM Tris-HCl, pH 8.0, 150 mM NaCl, 1 mM DTT, and 0.1 mg mL^−1^ bovine serum albumin (BSA). For inhibition studies, M^pro^ was incubated with various concentrations of GC376 or boceprevir (4-fold serial dilution series starting at 100 μM) for 1 h in the reaction buffer containing BSA prior to addition of the substrate to initiate the reaction. Fluorescence intensity was measured once per minute on a Tecan Spark 10M plate reader.

### Crystallography.

M^pro^ at 12 mg mL^−1^ was mixed with 2 mM GC376 in 20 mM Tris, pH 8.0, 150 mM NaCl, 1 mM DTT, and 2.5% DMSO and subjected to crystallization screening. The best diffracting crystals were obtained by sitting drop vapor diffusion method with a reservoir solution consisting of 0.2 M potassium thiocyanate, 20% polyethylene glycol 3350 (PEG 3350), and 0.1 M Bis-Tris propane buffer, pH 6.5. Crystals under this condition typically grew as a cluster of thick plates, from which single crystals could be isolated for data collection. X-ray diffraction data to ~1.7 Å resolution were collected at the Northeastern Collaborative Access Team (NE-CAT) beamline 24-ID-C of the Advanced Photon Source (Lemont, IL) and processed using XDS (61). The structure of the M^pro^-GC376 complex was determined by molecular replacement with Phaser (62), using a reported M^pro^ structure as a search model (PDB ID 7C8U). Iterative model building and refinement were performed using Coot (63) and Phenix (64), respectively. A summary of data collection and model refinement statistics is shown in Table S3. Similar to several other SARS2 M^pro^ crystal structures (13, 42, 65), our M^pro^ crystals in the C2 space group showed a homodimerized protease mediated via a crystallographic dyad symmetry. Clear electron density was observed for GC376, covalently linked to Cys145 in the active site. Notably, prior studies have shown GC376 binding in the M^pro^ active site in two distinct conformations, with the phenyl moiety making direct protein contacts or flipping out toward solvent (PDB IDs 6WTT [35], 7D1M and 7C6U [33], 7CBT [66], 7CB7 [67], 7JSU [68], and 6WTK [13]). Our high-resolution crystal structure of M^pro^-GC376 complex has the latter conformation (Fig. S3; PDB ID 6C8U). 

10.1128/mbio.00784-22.10 TABLE S3 X-ray data collection and model refinement statistics. Download Table S3, PDF file, 0.07 MB. Copyright © 2022 Moghadasi et al. 2022 Moghadasi et al. https://creativecommons.org/licenses/by/4.0/ This content is distributed under the terms of the Creative Commons Attribution 4.0 International license.

### RT-qPCR.

To quantify the relative levels of Src-M^pro^-Tat-eGFP mRNA relative to an RNA PolIII-driven control transcript, the U6-Luc-Src-M^pro^-Tat-eGFP plasmid (Fig. S5c) was transfected into 293T cells, and 24 h post-transfection, cells were recovered, and RNA was extracted from the cytosolic fraction. cDNA was synthesized using random hexamers, and qPCR was performed using SsoFast master mix (Promega; catalog no. 1725200). Primers are listed in Table S2. All reactions were run using a Roche LightCycler 480 instrument.

### Data availability.

The atomic coordinates and structure factors for our M^pro^-GC376 complex structure have been deposited in the RCSB Protein Data Bank with accession code PDB 7TGR (Table S3).
