# Supplementary figures and images for: Gain-of-Signal Assays for Probing Inhibition of SARS-CoV-2 Mpro/3CLpro in Living Cells
Source: mBio. 2022 Apr 26;13(3):e00784-22. doi: 10.1128/mbio.00784-22 (PMC9239272; doi:10.1128/mbio.00784-22)

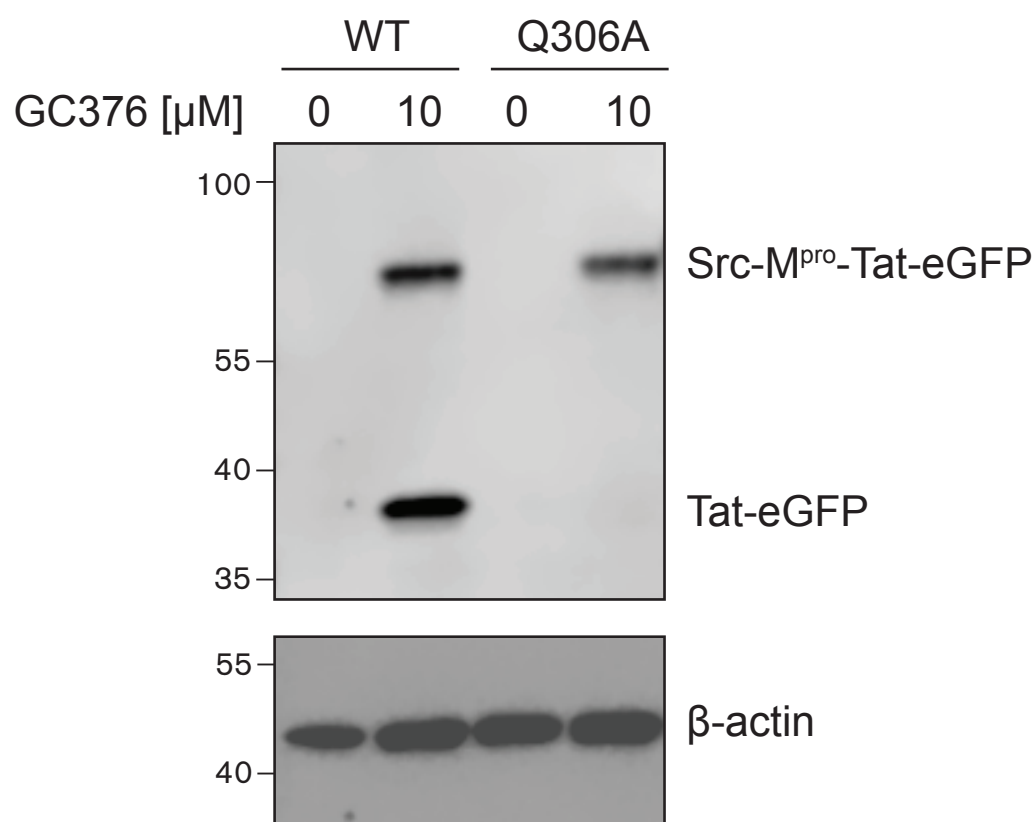

Supplement: FIG S1 [file mbio.00784-22-s0001.pdf]

a

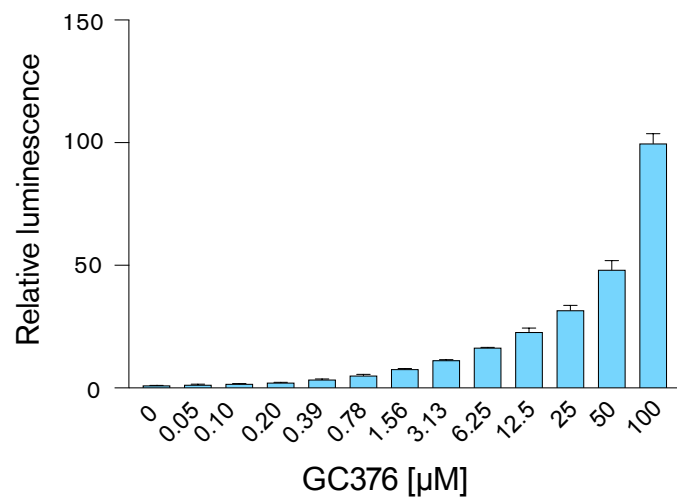

b

| Concentration [ $\mu\text{M}$ ] | Mean S:B |
|---------------------------------|----------|
| 0                               | 1        |
| 0.05                            | 1.3      |
| 0.10                            | 1.7      |
| 0.20                            | 2.2      |
| 0.39                            | 3.4      |
| 0.78                            | 5.0      |
| 1.56                            | 7.7      |
| 3.13                            | 11.3     |
| 6.25                            | 16.4     |
| 12.5                            | 22.8     |
| 25                              | 31.7     |
| 50                              | 48.2     |
| 100                             | 99.7     |

c

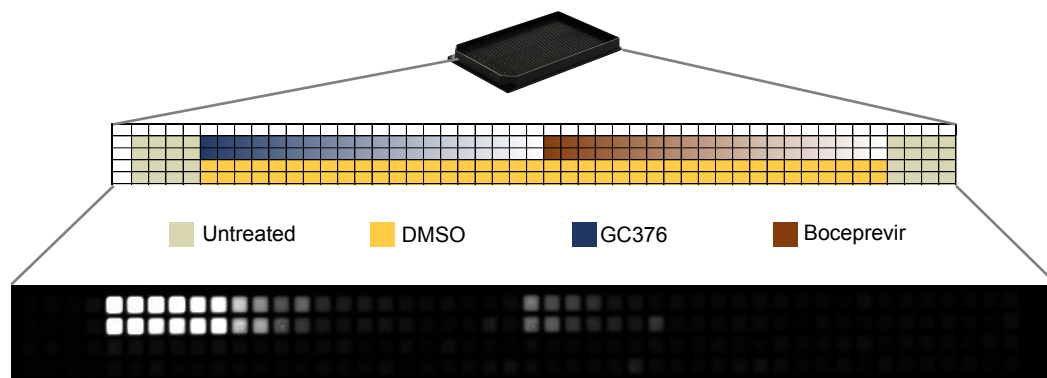

d

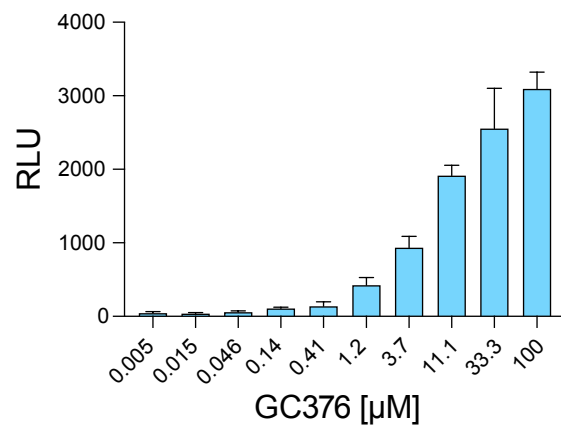

e

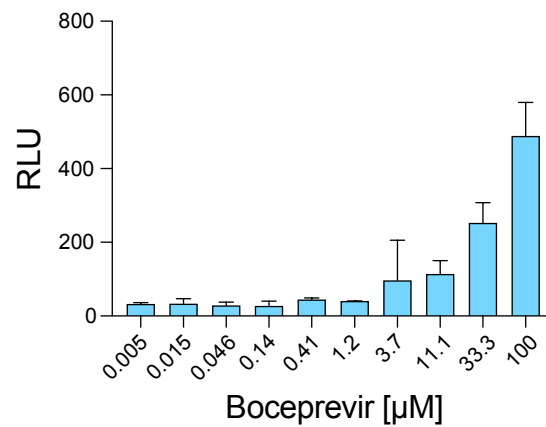

Supplement: FIG S2 [file mbio.00784-22-s0002.pdf]

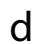

e

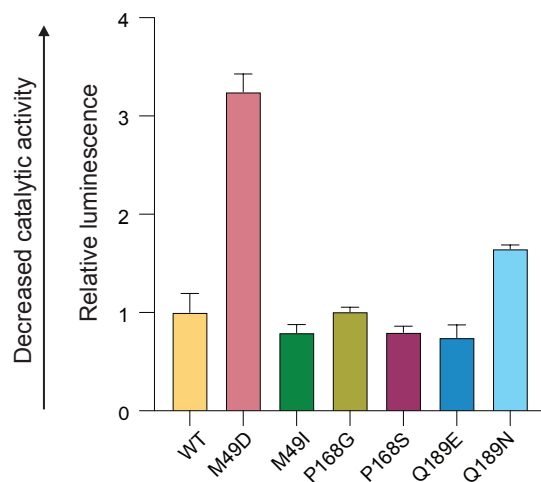

Supplement: FIG S3 [file mbio.00784-22-s0003.pdf]

a

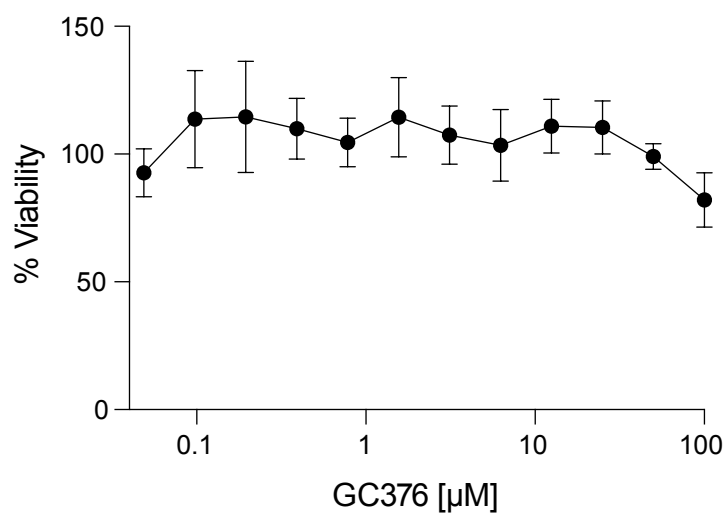

b

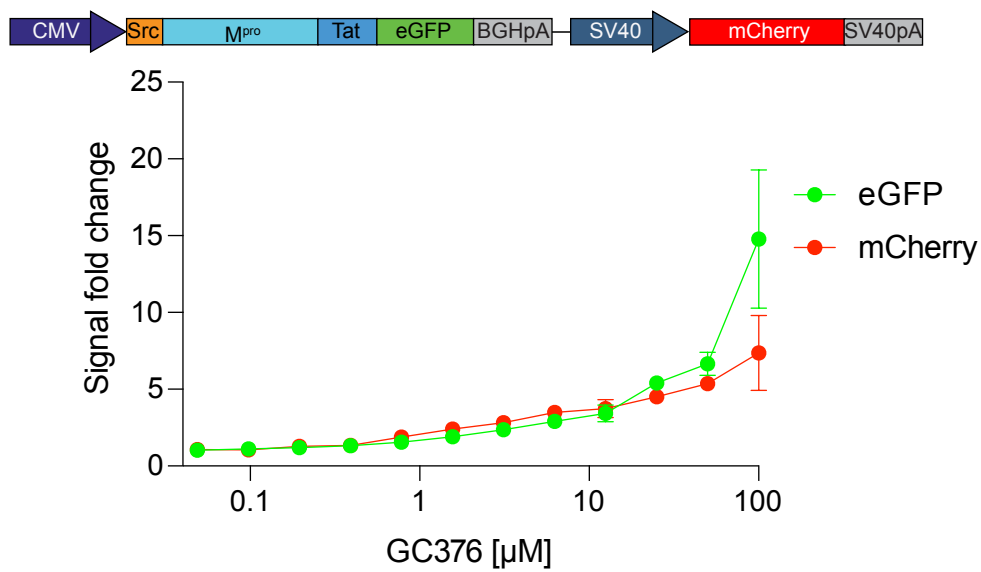

c

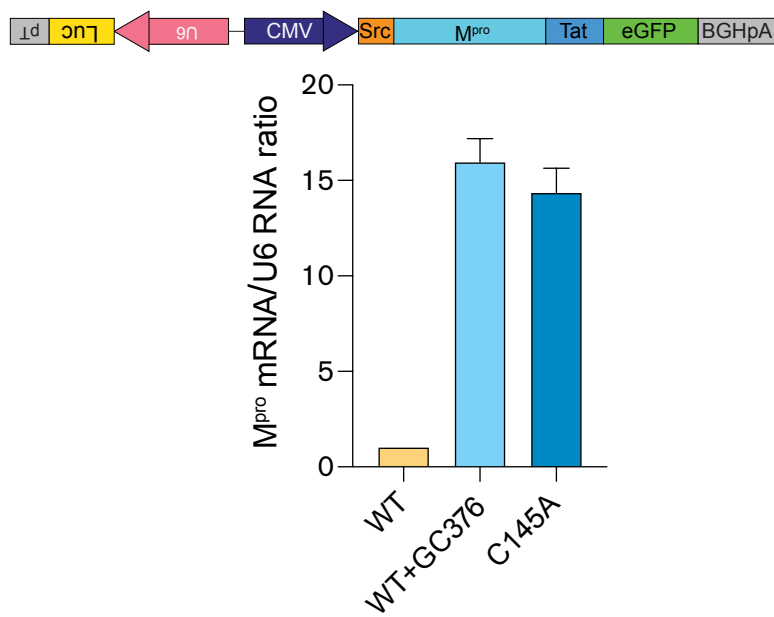

Supplement: FIG S5 [file mbio.00784-22-s0005.pdf]

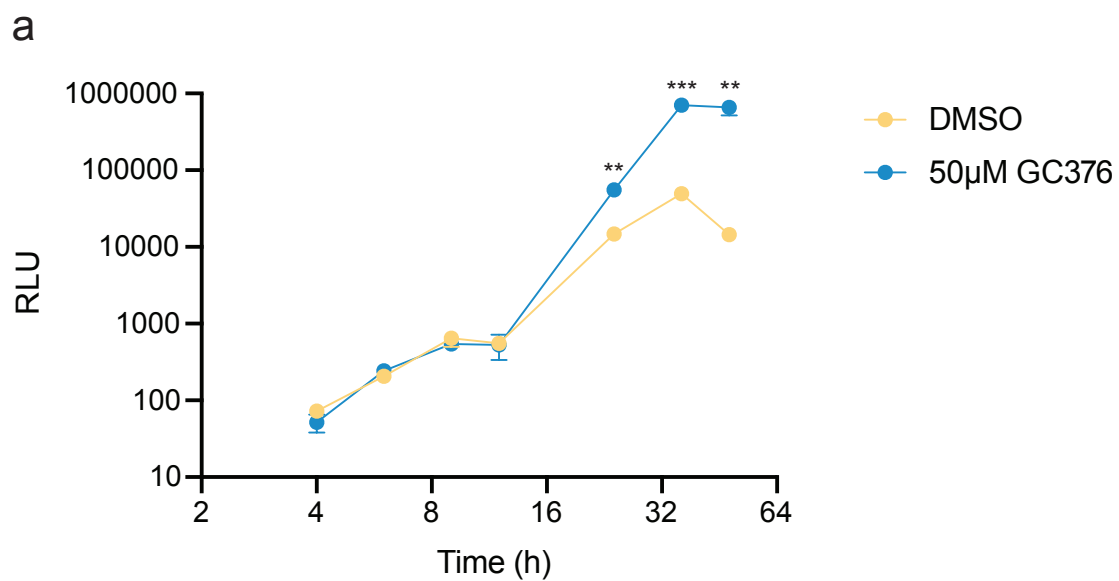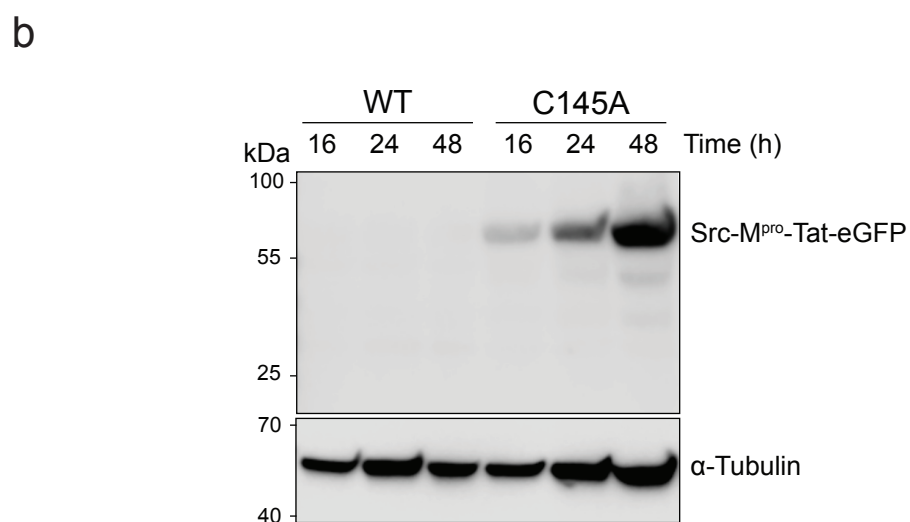

Supplement: FIG S4 [file mbio.00784-22-s0004.pdf]
