# Supplementary material for: Gain-of-Signal Assays for Probing Inhibition of SARS-CoV-2 Mpro/3CLpro in Living Cells
Source: mBio. 2022 Apr 26;13(3):e00784-22. doi: 10.1128/mbio.00784-22 (PMC9239272; doi:10.1128/mbio.00784-22)
Supplement: TABLE S1 [file mbio.00784-22-s0008.pdf]

Table S1 Inhibitor sources

Drug name	Vendor	MW	Purity	HPLC Trace
GC376	Selleckchem S0475	508	ND	ND
Boceprevir	Selleckchem S3733	520	ND	ND
Nirmatrelvir	MedChemExpress HY-138687	500	ND	ND
GRL-0496	MedChemExpress HY-137954Data	273	ND	ND
Carmofur	Selleckchem S1289	257	98%	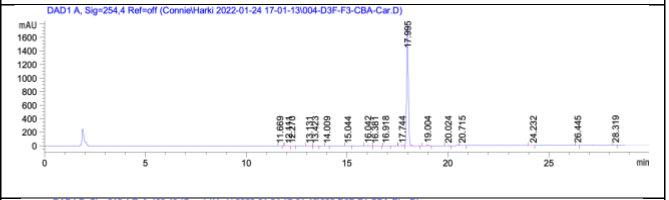
Ebselen	Selleckchem S6676	274	93%	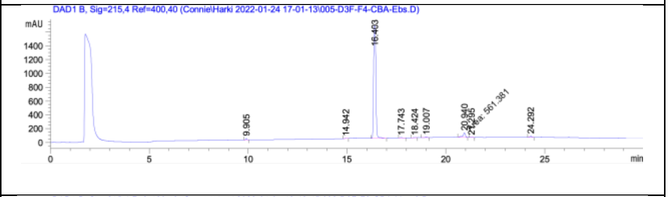
Masitinib	Selleckchem S1064	499	98%	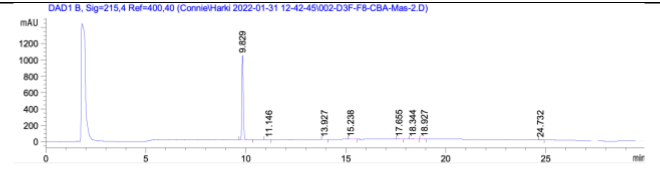
Ethacridine	Selleckchem S4196	343	99%	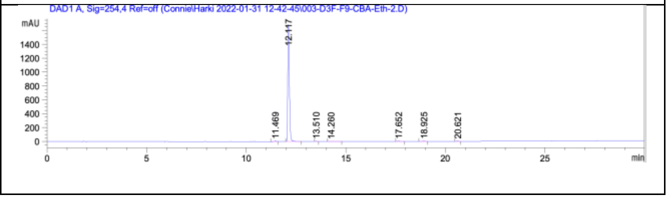
