# Supplementary material for: Gain-of-Signal Assays for Probing Inhibition of SARS-CoV-2 Mpro/3CLpro in Living Cells
Source: mBio. 2022 Apr 26;13(3):e00784-22. doi: 10.1128/mbio.00784-22 (PMC9239272; doi:10.1128/mbio.00784-22)
Supplement: FIG S6 [file mbio.00784-22-s0006.pdf]

a

Residue number	Domain description	GenBank accession number (matching residue numbers)
**1-10**	Src myristoylation motif	AAD13831.1 (1-10)
**11-15**	Linker	-
**16-337**	SARS2 M^{pro} with cognate N- and C-terminal cleavage sites	QNO91750.1 (3256-3577)
**338-346**	Linker	-
**347-418**	HIV-1 Tat	ACD75161.1 (1-72)
**419-424**	Linker	-
**425-663**	eGFP	AAB02576.1 (1-239)

Amino acid sequence:

MGSSKSKPKDGGGGSSITSAVLQSGFRKMAFPSGKVEGCMVQVTCGTTTLNGLWLDDVVYCPRHVICTSEDMLNPNYEDLLIRKSNHNFLVQAGNVQ
LRVIGHSMQNCVLKLKVDTANPKTPKYKFVRIQPGQTFSVLACYNGSPSGVYQCAMRPNFTIKGSFLNGSCGSVGFNIDYDCVSFCYMHHMELPTGV
HAGTDLEGNFYGPFVDRQTAQAAGTDTTITVNVLAWLYAAVINGDRWFLNRFTTTLNDFNLVAMKYNYEPLTQDHVDILGPLSAQTGIAVLDMCASL
KELLQNGMNGRTILGSALLEDEFTPFDVVRQCSGVTFQSAVKRTIKGTGSGGSGGMEPVDPRLEPWKHPGSQPKTACTNCYCKKCCFHCQVCFITKA
LGISYGRKKRRQRRRPPQGSQTHQVSLSKQPGSIATMVSKGEELFTGVVPILVELDGDVNGHKFSVSGEGEGDATYGKLTLKFICTTGKLPVPWPTL
VTTLTYGVQCFSRYPDHMKQHDFFKSAMPEGYVQERTIFFKDDGNYKTRAEVKFEGDTLVNRIELKGIDFKEDGNILGHKLEYNYNSHNVYIMADKQ
KNGIKVNFKIRHNIEDGSVQLADHYQQNTPIGDGPVLLPDNHYLSTQSALSKDPNEKRDHMVLLEFVTAAGITLGMDELYK*

b

Nucleotide sequence:

AAGCTTGCCACCATGGGCAGCAGTAAGAGTAAACCGAAAGATGGAGGCGGTGGGTCATCTATCACCTCAGCTGTTTTGCAGTCTGGTTTTAGGAAAA
TGGCGTTCCCCAGCGGTAAAGTTGAAGGATGTATGGTCCAAGTAACCTGTGGTACCACTACCCTTAATGGGCTTTGGTTGGACGACGTAGTCTACTG
CCCCCGACACGTAATCTGCACTAGTGAGGATATGCTTAATCCCAATTACGAAGACCTTTTGATTCGGAAATCCAATCACAACTTCCTGGTCCAAGCG
GGCAACGTCCAACTCAGGGTTATTGGACATAGTATGCAGAATTGCGTACTGAAGCTCAAAGTCGATACTGCAAACCCCAAGACGCCCAAGTATAAAT
TCGTCCGAATCCAACCAGGCCAAACATTTTCCGTATTGGCTTGCTATAATGGAAGCCCCAGCGGTGTCTACCAATGTGCAATGAGACCAAACTTTAC
GATAAAGGGTTCATTTCTGAACGGCTCTTGCGGTTCCGTTGGTTTTAACATCGACTATGACTGTGTATCCTTTTGCTACATGCACCATATGGAACTC
CCTACCGGTGTCCACGCCGGTACAGATCTGGAAGGAAATTTCTACGGTCCGTTCGTTGACCGGCAAACCGCGCAAGCGGCTGGAACCGACACAACGA
TTACAGTGAATGTGCTCGCGTGGCTGTACGCAGCAGTCATAAACGGAGACAGGTGGTTTCTGAACCGATTTACGACGACTCTCAATGACTTCAACCT
TGTTGCGATGAAGTACAATTACGAGCCACTCACCCAGGACCATGTTGATATCCTGGGTCCCCTCAGTGCCCAGACAGGGATCGCAGTTCTCGATATG
TGCGCGTCACTGAAGGAGCTTCTCCAAAATGGAATGAATGGGCGGACCATACTTGGTTCCGCACTCCTCGAAGATGAATTTACTCCATTTGACGTGG
TCAGACAATGCAGTGGGGTCACTTTCCAGAGTGCAGTGAAAAGAACAATCAAGGGTACCGGGTCAGGGGGCAGCGGAGGGATGGAGCCAGTAGATCC
TAGACTAGAGCCCTGGAAGCATCCAGGAAGTCAGCCTAAAACTGCTTGTACCAATTGCTATTGTAAAAAGTGTTGCTTTCATTGCCAAGTTTGTTTC
ATAACAAAAGCCTTAGGCATCTCCTATGGCAGGAAGAAGCGGAGACAGCGACGAAGACCTCCTCAAGGCAGTCAGACTCATCAAGTTTCTCTATCAA
AGCAGCCGGGATCCATCGCCACCATGGTGAGCAAGGGCGAGGAGCTGTTCACCGGGGTGGTGCCCATCCTGGTCGAGCTGGACGGCGACGTAAACGG
CCACAAGTTCAGCGTGTCCGGCGAGGGCGAGGGCGATGCCACCTACGGCAAGCTGACCCTGAAGTTCATCTGCACCACCGGCAAGCTGCCCGTGCCC
TGGCCCACCCTCGTGACCACCCTGACCTACGGCGTGCAGTGCTTCAGCCGCTACCCCGACCACATGAAGCAGCACGACTTCTTCAAGTCCGCCATGC
CCGAAGGCTACGTCCAGGAGCGCACCATCTTCTTCAAGGACGACGGCAACTACAAGACCCGCGCCGAGGTGAAGTTCGAGGGCGACACCCTGGTGAA
CCGCATCGAGCTGAAGGGCATCGACTTCAAGGAGGACGGCAACATCCTGGGGCACAAGCTGGAGTACAACTACAACAGCCACAACGTCTATATCATG
GCCGACAAGCAGAAGAACGGCATCAAGGTGAACTTCAAGATCCGCCACAACATCGAGGACGGCAGCGTGCAGCTCGCCGACCACTACCAGCAGAACA
CCCCCATCGGCGACGGCCCCGTGCTGCTGCCCGACAACCACTACCTGAGCACCCAGTCCGCCCTGAGCAAAGACCCCAACGAGAAGCGCGATCACAT
GGTCCTGCTGGAGTTCGTGACCGCCGCCGGGATCACTCTCGGCATGGACGAGCTGTACAAGTAAAGCGGCCGC
