# Supplementary material for: Gain-of-Signal Assays for Probing Inhibition of SARS-CoV-2 Mpro/3CLpro in Living Cells
Source: mBio. 2022 Apr 26;13(3):e00784-22. doi: 10.1128/mbio.00784-22 (PMC9239272; doi:10.1128/mbio.00784-22)
Supplement: TABLE S2 [file mbio.00784-22-s0009.pdf]

Table S2 Site directed mutagenesis and qPCR primers

**Primer Name**	Sequence
M^{pro} M49D forward	GCACTAGTGAGGATGACCTTAATCCCAATTACGAAGACC
M^{pro} M49D reverse	GTAATTGGGATTAAGGTCATCCTCACTAGTGCAGATTACGTG
M^{pro} M49I forward	GCACTAGTGAGGATATTCTTAATCCCAATTACGAAGACC
M^{pro} M49I reverse	GTAATTGGGATTAAGAATATCCTCACTAGTGCAGATTACGTG
M^{pro} P168G forward	GGAACTCGGTACCGGTGTCCACGCCG
M^{pro} P168G reverse	GGACACCGGTACCGAGTTCCATATGGTGCATG
M^{pro} P168S forward	TGGAACTCTCAACCGGTGTCCACGC
M^{pro} P168S reverse	CACCGGTTGAGAGTTCCATATGGTGCATGTAGC
M^{pro} Q189E forward	TGACCGGGAAACCGCGCAAGCG
M^{pro} Q189E reverse	TTGCGCGGTTTCCCGGTCAACGAAC
M^{pro} Q189N forward	TGACCGGAACACCGCGCAAGCG
M^{pro} Q189N reverse	TTGCGCGGTGTTCCGGTCAACGAAC
M^{pro} H41A forward	CCCCGAGCGGCAATCTGCACTAGTGAGGATATGC
M^{pro} H41A reverse	GCCCCCGAGCGGTAATCTGCACTAGTGAGGATATGC
M^{pro} S10A forward	AGTGCAGATTACCGCTCGGGGGCAGTAGACTACG
M^{pro} S10A reverse	CTTCAACTTTACCTGCGGGGAACGCCATTTTCCTAAAACC
M^{pro} E14A forward	CGGTAAAGTTGCAGGATGTATGGTCCAAGTAACC
M^{pro} E14A reverse	ACCATACATCCTGCAACTTTACCGCTGG
M^{pro} E290A forward	TCCTCGAAGATGCCTTTACTCCATTTGACGTGGTCAG
M^{pro} E290A reverse	AAATGGAGTAAAGGCATCTTCGAGGAGTGCG
M^{pro} qPCR forward	GCTCAAAGTCGATACTGCAAAC
M^{pro} qPCR reverse	GCTTCCATTATAGCAAGCCAATAC
Luc qPCR forward	GCTATGAAGAGATACGCCCTG
Luc qPCR reverse	TAGCTTCTGCCAACCGAAC
