# Supplementary material for: Gain-of-Signal Assays for Probing Inhibition of SARS-CoV-2 Mpro/3CLpro in Living Cells
Source: mBio. 2022 Apr 26;13(3):e00784-22. doi: 10.1128/mbio.00784-22 (PMC9239272; doi:10.1128/mbio.00784-22)
Supplement: TABLE S3 [file mbio.00784-22-s0010.pdf]

Table S3 X-ray data collection and model refinement statistics

	M^{Pro}/GC376 (7tgr)
Data collection	
Resolution range (Å)	47.85 - 1.68 (1.72 - 1.68)
Space group	*C*2
Unit cell	
(*a*, *b*, *c* in Å)	114.56, 52.94, 45.90
(α, β, γ in °)	90, 102.551, 90
Total reflections	111667 (7620)
Unique reflections	30122 (1993)
Multiplicity	3.7 (3.8)
Completeness (%)	97.44 (96.19)
Mean $I/\sigma(I)$	11.36 (1.06)
R_{merge}	0.0636 (1.23)
R_{meas}	0.0744 (1.43)
R_{pim}	0.0382 (0.719)
$CC_{1/2}$	0.998 (0.526)
Refinement	
No. of Reflections	29949 (1992)
No. of Refl. for R_{free}	1505 (94)
R_{work}	0.179 (0.323)
R_{free}	0.228 (0.347)
Number of non-H atoms	2579
Macromolecules	2406
Ligands	20
Solvent	167
Average B-factor ($Å^2$)	39.03
Macromolecules	38.54
Ligands	53.84
Solvent	45.66
Protein residues	305
R.m.s.d.	
Bond length (Å)	0.018
Bond angles (°)	1.57
Ramachandran plot	
Favored (%)	98.01
Allowed (%)	1.99
Outliers (%)	0.00

Statistics for the highest-resolution shell are shown in parentheses.
